# Prevalence of dementia in patients seen at a private hospital in the Southern Region of Brazil

**DOI:** 10.31744/einstein_journal/2020AO4752

**Published:** 2019-10-17

**Authors:** Ricardo Krause Martinez de Souza, Amanda Ferraz Barboza, Graciany Gasperin, Heloize Dzieciol Berthier Portes Garcia, Paola Martins Barcellos, Renato Nisihara

**Affiliations:** 1 Ambulatório de Desordens da Memória e do Comportamento, Instituto de Neurologia de Curitiba, Curitiba, PR, Brazil.; 2 Universidade Positivo, Curitiba, PR, Brazil.

**Keywords:** Dementia, Alzheimer disease, Cognitive dysfunction

## Abstract

**Objective:**

To evaluate the epidemiological profile of patients seen at a dementia outpatient clinic.

**Methods:**

A retrospective study conducted by medical record review searching data on sex, race, age, schooling level, and diagnosis of patients seen from 2008 to 2015.

**Results:**

A total of 760 patients were studied, with a predominance of female (61.3%; p<0.0001). The mean age was 71.2±14.43 years for women and 66.1±16.61 years for men. The most affected age group was 71 to 80 years, accounting for 29.4% of cases. In relation to race, 96.3% of patients were white. Dementia was diagnosed in 68.8% of patients, and Alzheimer’s disease confirmed in 48.9%, vascular dementia in 11.3%, and mixed dementia in 7.8% of cases. The prevalence of dementia was 3% at 70 years and 25% at 85 years. Dementia appeared significantly earlier in males (mean age 68.5±15.63 years). As to sex distribution, it was more frequent in women (59.6%) than in men (40.4%; p<0.0001; OR=2.15). People with higher schooling level (more than 9 years) had a significantly younger age at onset of dementia as compared to those with lower schooling level (1 to 4 years; p=0.0007).

**Conclusion:**

Most patients seen in the period presented dementia, and Alzheimer was the most prevalent disease. Women were more affected, and men presented young onset of the disease. Individuals with higher schooling level were diagnosed earlier than those with lower level.

## INTRODUCTION

Dementia has become a huge public health problem because of the increased life expectancy of the world population, which has direct social and economic impact in the structure of the society.^1^ Dementia causes suffering for patients and families, especially due to progressive impairment and dependence on other individuals to maintain the Activities of Daily Life.^2^

Alzheimer’s disease is the most common cause of dementia, accounting for approximately 60 to 80% of cases,^2^ followed by vascular dementia (20% of cases).^1^ Burlá et al.,^3^ carried out a literature review between 1995 and 2012 and estimated the prevalence of dementia in Brazil as 7.6% in individuals aged 65 years or older. In 2016, global data showed 438 million people with dementia (95% confidence interval − 95%CI: 37.8-51.0).^4^

Dementia increases exponentially as from the age of 60 years, and its prevalence doubles every 5 years.^5^ One percent of 60-year-old individuals have dementia, and the prevalence at 85 years ranges from 20 to 30%.^5^ Some projections estimate that by 2020, 15% of the Brazilian population will be over 60 years, and Brazil will rank sixth in number of elderly, in 2025.^3^

Mild cognitive impairment (MCI) is characterized by cognitive complaint reported by the patient and/or family member, and by objective cognitive impairment assessed by cognitive tests, based on age-related and schooling parameters. In addition, functional activities must be preserved.^6^ Some studies consider MCI as a transition between cognitive changes observed in aging and dementia.^7^ Therefore, it is important to classify MCI into subtypes, namely: amnestic MCI single domain (aMCI-SD), amnestic MCI multiple domain (aMCI-MD), non-amnestic MCI single domain (naMCI-SD), and non-amnestic MCI multiple domain (naMCI-MD).^8^ When the amnestic MCI subtype progresses to dementia, Alzheimer’s disease is the most common condition. In the case of non-amnestic MCI, progression to frontotemporal lobar degeneration or dementia with Lewy bodies is more frequent.^7^ Prevalence data of MCI in the international literature varies from 3 to 42%.^9^

## OBJECTIVE

To evaluate the epidemiologic profile of patients referred with suspected mild cognitive impairment or dementia to a reference outpatient clinic for memory and behavior disorders.

## METHODS

This study was submitted to and approved by the Institutional Review Board of the *Universidade Positivo* under number 1.533.211, CAAE: 55276416.0.0000.0093. This is a retrospective study, carried out by medical record review, using an electronic database provided by the *Instituto de Neurologia de Curitiba* , in the city of Curitiba (State of Paraná). The study collected data from patients referred to the outpatient clinic between January 2008 and December 2015.

Patients aged ≥18 years who met the diagnostic criteria for dementia, according to the Diagnostic and Statistical Manual of Mental Disorders, fifth edition (DSM-IV), of the American Psychiatric Association (APA),^10^ and/or diagnostic criteria for MCI were included.^11^

The exclusion criteria were incomplete medical records or diseases that affected cognition that did not meet criteria for dementia syndrome or MCI, such as: attention deficit hyperactivity disorder, traumatic brain injury, neurodevelopmental diseases, vascular cognitive impairment, no dementia (VCI-ND), and cognitive changes related to epilepsy.

All patients were evaluated by the same team of neurologists. The investigation carried out was part of the outpatient care protocol and complied with the recommendations of the Scientific Department on Cognitive Neurology and Aging of the Brazilian Academy of Neurology.^11^ The investigation consisted of detailed history, clinical and neurological examination, cognitive assessment, blood tests, and neuroimaging - preferably brain magnetic resonance (MRI) or brain computed tomography (CT), when brain MRI was contraindicated.

The data collected for this study were sex, race, age, schooling level, and hand dexterity. Diagnosis was established based on the diagnostic criteria of the *Ambulatório de Desordens da Memória e do Comportamento* (ADEMEC) protocol. Specific diagnostic criteria were therefore used for each type of dementia, as follows: National Institute of Neurological and Communicative Disorders and Stroke (NINDS), and Alzheimer’s Disease and Related Disorders Association, for AD;^12^ NINDS and *Association Internationale pour la Recherche et l’Enseignement en Neurosciences* (AIREN), for vascular dementia;^13^ Third Report of the Dementia Lewy Bodies (DLB) Consortium, for Lewy bodies Dementia;^14^ and Lund and Manchester criteria for frontotemporal dementia.^15^

According to the diagnosis, the patients included were divided into three groups: dementia syndrome, MCI, and other rare dementia syndromes.

The Clinical Dementia Rating (CDR) scale^16 , 17^ was the instrument used to assist in clinical diagnosis and to classify the stage of dementia. In addition, CDR has been used in clinical practice to help differentiate patients with MCI from those who progress to dementia.^17^ A score equal to zero corresponds to no dementia; 0.5 is uncertain or corresponds to MCI; 1 to mild dementia; 2 to moderate dementia, and 3 to severe dementia.

The collected data were spread using Excel^®^ software and statistical analysis was conducted with the aid of Graph Pad Prism 6.0 software (La Jolly, USA). Continuous variables were expressed as mean±standard deviation and compared using the *t* and Mann-Whitney tests. Categorical variables were expressed as percentages and compared by the χ^2^ test or Fisher’s exact test as appropriate; p values, odds ratio (OR), and 95%CI were calculated. Values of p<5% were considered significant.

## RESULTS

The medical records of 1,060 patients referred to the Memory and Behavior Disorder Outpatient Clinic during the study period were evaluated. Of the 1,060 patients evaluated, 760 patients were included. The main reason for excluding patients was that the outpatient clinic initially treated patients with different cognitive complaints. Patients were referred to other specialized outpatient clinics only after establishing the diagnosis of cognitive disorders not compatible with dementia syndrome and/or MCI.

The patients included (n=760) were divided into three groups: dementia syndromes, MCI, and other rare dementia syndromes. There was a predominance of dementia, accounting for 68.8% (523/760) of the cases studied. Mild cognitive impairment was diagnosed in 17.4% (133/760), and rare degenerative and/or non-degenerative dementia accounted for 13.8% (102/760) of patients.

Demographic data of the patients studied are depicted in table 1 . Among the referred patients, there was a significant predominance of females (61.3%; p<0.0001; OR=2.5; 95%CI: 2.0-3.1). The mean age was 67.7±15.67 years. As presented in table 1 , there was a significant increase in the number of cases in those aged over 60 years. Regarding race, 96.3% were white, and 59.5% of patients had complete or incomplete higher education.

**Table 1:** Demographic data of patients studied (n=760)

	n (%)
Sex	
Female	467 (61.3)
Male	293 (38.5)
Age, years	
18 - 30	23 (3)
31 - 40	32 (4.2)
41 - 50	56 (7.3)
51 - 60	101 (12.2)
61 - 70	152 (20.0)
71 - 80	224 (29.4)
81 - 90	156 (20.5)
91 - 100	16 (2.1)
Race	
White	732 (96.3)
Black	16 (2.1)
Yellow	7 (0.9)
Indigenous	5 (0.6)
Schooling level, years	
Illiterate	11 (1.3)
0	18 (2.3)
1 - 4	228 (30)
5 - 8	50 (6.5)
9 - 11	131 (17.2)
> 11	322 (42.3)

Table 2 depicts the dementia types found when the groups were analyzed separately. Alzheimer’s disease represented almost half of the cases of dementia (48.9%), followed by vascular dementia (11.3%), and mixed dementia (7.8%), which were found in patients with Alzheimer’s disease and concomitant vascular dementia. Dementia was more frequent in women (312/523; 59.6%) than in men (211/523; 40.4%) with significant difference (p<0.0001; OR=2.1; 95%CI: 1.6-2.7). The mean age of patients was 70.1±14.90 years. The mean age of men was 68.5±15.63 years, and women, 71.2±14.43 years; men had dementia at a younger age than women (p<0.0001).

**Table 2:** Classification of dementia syndromes per prevalence (n=523)

Dementia syndromes	n (%)
Alzheimer’s disease	256 (48.9)
Vascular dementia	59 (11.3)
Mixed dementia	41 (7.8)
Normal pressure hydrocephalus	31 (5.9)
Lewy bodies dementia	26 (5)
Unspecific dementia	21 (4)
Progressive supranuclear palsy	15 (2.9)
Frontotemporal dementia	12 (2.3)
Others	62 (11.9)

Regarding schooling level and age at diagnosis, when comparing people with higher schooling (over 9 years) to those with less (1 to 4 years), the age of onset of dementia was significantly lower in those with higher schooling levels (p=0.0007). When comparing those with higher schooling (over 9 years) with those with medium schooling level (5 to 8 years), no significant difference was observed (p=0.09). The difference between the individuals with 1 to 4 years and those with 5 to 8 years of schooling was statistically significant (p=0.036).

Table 3 shows the distribution of CDR scores, which assessed the dementia stage of the patients studied. When comparing Alzheimer’s disease and vascular dementia, there was no significant difference between the CDR stages (p=0.78), hence severity was similar among the most commonly found dementias. Correlating the influence of schooling level with CDR, in patients with lower schooling (less than 9 years of school), CDR had significantly higher score as compared to patients with higher schooling level (more than 9 years; p<0.0001; OR=3.6; 95%CI: 2.4-5.3). It is worth mentioning that CDR stage 3 was frequent in more patients with lower schooling levels.

**Table 3:** Analysis of stages of the Clinical Dementia Rating Scale in patients with dementia, comparing type of dementia and schooling

	CDR=1	CDR=2	CDR=3
	n (%)	n (%)	n (%)
Type of dementia			
Alzheimer’s disease (n=256)	119 (46.5)	95 (37.1)	42 (16.4)
Vascular dementia (n=59)	24 (40.7)	28 (47.4)	7 (11.9)
Others (n=208)	84 (40.7)	82 (39.4)	42 (19.9)
Schooling*			
Up to 9 years (n=196)*	57 (29.0)	89 (45.4%)	50 (25.5)
More than 9 years (n=263)	157 (59.7)	79 (30.0)	26 (9.8)

* Comparing schooling level *versus* CDR=3 (p<0.0001; OR=3.6; 95%CI: 2.4-5.3). CDR: Clinical Dementia Rating Scale.

As for CDR staging, upon diagnosis, 46.5% of patients were in stage 1; 37.0% stage 2, and 16.5% stage 3.

Regarding patients diagnosed with MCI, on table 4 we see the types of MCI found. Among the five subtypes, differentiated according to the type of domain affected, the aMCI-SD subtype was the most prevalent, found in 77.3% of cases. In relation to the age of patients with MCI, the mean age for patients was 61.7±14.82 years; and 59.1±15.74 years for men, and 62.2 ± 16.02 years for women. When comparing the meam ages, there was no difference in age of onset of MCI between men and women.

**Table 4:** Classification of patients with mild cognitive impairment according to the subtype (n=133)

Types of MCI	n (%)
aMCI-SD	103 (77.3)
aMCI-MD	18 (13.6)
naMCI-MD	6 (4.5)
MCI-IPD	2 (1.5)
naMCI-SD	2 (1.5)
Cognitive impairment to be determined	2 (1.5)

MCI: mild cognitive impairment; aMCI-SDa: amnestic MCI single-domain; aMCI-MD: amnestic MCI multiple domain; naMCI-MD: non-amnestic MCI multiple domain; IPD: idiopathic Parkinson’s disease; naMCI-SD: non-amnestic MCI single domain.

Regarding schooling, patients with more school years (≥9 years) presented MCI significantly earlier as compared to those with less than 9 years of school (p=0.03).

## DISCUSSION

This study presents in an original way the epidemiology of a robust sample of Brazilian health insured or private patients with dementia and MCI, treated in a referral center in southern Brazil. These patients are differentiated for having high schooling and income levels, when compared to the population of the country.

Regarding sex, there was a higher prevalence of dementia among women, twice as frequent as in men, corroborating data obtained by other authors.^1 , 3^ One possible explanation for the higher prevalence of dementia in women may be life expectancy. Additionally, some factors such as greater exposure to work-related stress, alcohol and cigarette abuse, may also play a relevant role in the greater prevalence of dementia in women.^3^ Bottino et al., however, found no difference between men and women in the prevalence of dementia.^18^

As to age-related classification, early onset (presenile) dementia, with onset of cognitive symptoms in people aged under 65 years, was more frequent in men. Some studies have also found a predominance of early-onset dementia in males.^19 , 20^ Lifestyle habits classically associated to men, such as alcoholism and smoking, among others, may influence this finding.

In this study, among all patients diagnosed with dementia or MCI, there was a significant increase in prevalence in the 61 to 70-year-old age group (20%), 71 to 80 (29%), and 81 to 90 (20%). However, in the elderly aged between 91 and 100 years, it was 2%. Some studies with patients at very advanced ages - above 90 years - suggested the occurrence of a plateau. Nonetheless, the number of individuals aged over 90 years in our study was too small to allow such an assessment. This association between age and increased prevalence of dementia or MCI has also been found by other authors in the Brazilian population.^18 , 21 , 22^

In this study, dementia affected white individuals in 96.3% of cases. Lopes et al.,^23^ showed no differences in the prevalence of dementia between black and white patients. We emphasize the inequal income distribution prevailing in our country enables greater access of white individuals to private care, and the Southern Region was predominantly colonized by Caucasians - a fact that does not reflect the reality in most regions of Brazil.

Higher schooling levels were significantly associated with earlier onset of dementia. This finding is inconsistent with the literature.^18 , 22 , 24^ In the Brazilian population, individuals with lower education have a higher risk of dementia, up to six-fold higher in illiterate people.^18 , 22 , 24^ Our research was conducted in a private hospital with a group of patients with higher schooling and income than the general Brazilian population - only 1.1% of patients were illiterate. We believe we presented a profile of middle-class Brazilians, whose data may differ from those observed in studies conducted with patients seen by the Brazilian National health System (SUS - *Sistema Único de Saúde* ). One possibility may be that patients with higher schooling performed diversified daily activities and were subject to greater cognitive demand, and the small cognitive changes were more easily perceived. In addition, higher income enables easier and faster access to outpatient clinics that provide care for this demand, a fact that may also provide results different from those found in the literature.

As expected, Alzheimer’s disease was the most common form of dementia among our patients (48.9%), consonant with other authors.^25^ Rizzi et al.,^1^ described that Alzheimer’s disease accounted for 60% and vascular dementia for 20% of dementia cases, in different regions of the world.

There was no difference in CDR stage when the most common types of dementia were compared. However, almost half of the patients diagnosed with dementia had CDR 1 (mild) or 2 (moderate), and 16.5% sought care with CDR stage 3 (severe) – this data differentiated the private outpatient clinic profile of this study. Most patients sought specialized care in early stages of dementia. This is extremely relevant, since early diagnosis may result in a more favorable progress of the disease, when some measures are adopted, such as adequate control of cardiovascular risk factors; lifestyle changes including physical activity, avoiding certain medications that affect cognition, alcohol abuse, smoking, and cognitive rehabilitation; as well as early pharmacological treatment. In addition, early diagnosis of the disease allows the patient, family members and caregivers to be better prepared to deal with the disease and its progression.^1 - 4^

Clinical Dementia Rating stage 3 was diagnosed in lower schooling individuals three times more often than in those with higher education levels. This fact may be due to the greater delay of less educated people to seek a specialized service. A study conducted in the Brazilian state of Rio Grande do Sul^26^ using the same score found no influence of patients’ schooling level in the CDR classification.

The prevalence data on MCI in the international literature are extremely variable, due to the different diagnostic criteria used.^9^ Mild cognitive impairment was diagnosed in 17.4%, with a mean age of 62.2 years, earlier than in the cases of dementia. Petersen et al., described that the prevalence of MCI was similar to that found in our study, ranging from 12 to 18%.^27^

Likewise dementia, MCI was more frequent in males than in females, and its onset was significantly earlier in patients with higher schooling (≥9 years). Among the four main MCI subtypes described, the most common is the amnestic type, however there are many different results in the international literature.^28^ It is worth mentioning that it is difficult to compare studies on prevalence of MCI, since different findings may be due to the use of diverse diagnostic criteria, and to influence of some factors, such as age, schooling level, instruments used, and genetics of the studied population.

The limitation of our study is the retrospective design, with information collected from medical records, what does not allow assessing the outcome of the population studied. Such data would be of great importance, since there is no further information on patients diagnosed with cognitive impairment no dementia. The concept of cognitive impairment no dementia encompasses patients with MCI and individuals with cognitive performance lower than expected for age and education, who may or may not decline.^29 , 30^ The strength of this study lies in the sample size and differentiated profile, including only patients from a private organization with high educational level, providing relevant data about a population very little studied in our country.

## CONCLUSION

Most patients evaluated had diagnosis of dementia. The most common dementia syndrome was Alzheimer’s disease. Regarding mild cognitive impairment, the amnestic single domain subtype was the most prevalent. Women were twice as affected as men, but men had significantly earlier onset. Patients with higher schooling level were diagnosed significantly younger than those with less education, both in relation to dementia and mild cognitive impairment. However, patients with less education had worse cognitive impairment.
