# Supplementary material for: Prevalence of dementia in patients seen at a private hospital in the Southern Region of Brazil
Source: Einstein (Sao Paulo). 2019 Oct 17;18:eAO4752. doi: 10.31744/einstein_journal/2020AO4752 (PMC6896655; doi:10.31744/einstein_journal/2020AO4752)
Supplement: Translation

# Translation: Prevalência de demência em pacientes atendidos em um hospital privado no sul do Brasil

**Authors:** Ricardo Krause Martinez de Souza, Amanda Ferraz Barboza, Graciany Gasperin, Heloize Dzieciol Berthier Portes Garcia, Paola Martins Barcellos, Renato Nisihara

**Affiliations:** 1 Ambulatório de Desordens da Memória e do Comportamento, Instituto de Neurologia de Curitiba, Curitiba, PR, Brasil.; 2 Universidade Positivo, Curitiba, PR, Brasil.

**Keywords:** Demência, Doença de Alzheimer, Disfunção cognitiva

## Abstract

**Objetivo:**

Avaliar o perfil epidemiológico dos pacientes atendidos em um ambulatório de demência.

**Métodos:**

Estudo retrospectivo realizado pela revisão de prontuários com busca de dados sobre sexo, raça, idade, escolaridade e diagnóstico de pacientes atendidos de 2008 a 2015.

**Resultados:**

Foram estudados 760 pacientes, com predomínio do sexo feminino (61,3%; p<0,0001). A média de idade foi 71,2±14,43 anos, para as mulheres, e de 66,1±16,61 anos, para os homens. A faixa etária mais acometida foi de 71 a 80 anos, representando 29,4% dos casos. Em relação à etnia, 96,3% dos pacientes eram brancos. O quadro de demência foi diagnosticado em 68,8% dos pacientes, tendo sido a doença de Alzheimer confirmada em 48,9% dos casos, demência vascular em 11,3% e mista em 7,8%. A prevalência de demência foi de 3% aos 70 anos e de 25% aos 85 anos. O quadro demencial apareceu significativamente mais cedo no sexo masculino (média de idade 68,5±15,63 anos). A distribuição entre os sexos mostrou demência mais frequente em mulheres (59,6%) do que em homens (40,4%; p<0,0001; RC=2,15). Pessoas com maior escolaridade (acima de 9 anos) apresentaram idade de aparecimento da demência significativamente menor que aquelas de menor escolaridade (1 a 4 anos; p=0,0007).

**Conclusão:**

Os pacientes atendidos no período apresentaram demência na maioria dos casos, sendo Alzheimer a doença mais prevalente. Mulheres foram mais acometidas, entretanto homens apresentaram a doença mais precocemente. Indivíduos com maior escolaridade foram diagnosticados mais cedo que aqueles com menor escolaridade.

## INTRODUÇÃO

A demência tornou-se grande problema de saúde pública devido ao aumento na expectativa de vida da população mundial. Isso incide diretamente na estrutura da sociedade, tanto no aspecto econômico quanto social.^1^ A demência causa sofrimento ao paciente e aos familiares, principalmente pela progressiva incapacidade e pela dependência de outros para manutenção de Atividades da Vida Diária.^2^

A doença de Alzheimer é a causa mais comum de demência, acometendo aproximadamente 60 a 80% dos casos,^2^ seguida da demência vascular, correspondendo a 20% dos casos.^1^ Burlá et al.,^3^ realizaram uma revisão de literatura entre 1995 a 2012 e estimaram a prevalência de demência no Brasil de 7,6% em pessoas com 65 anos ou mais. Dados mundiais mostram que, em 2016, o número global de indivíduos que viviam com demência era de 43,8 milhões (intervalo de confiança de 95% − IC95%=37,8-51,0).^4^

A demência aumenta de forma exponencial a partir dos 60 anos de idade, sendo que sua prevalência dobra a cada 5 anos.^5^ Representa 1% dos idosos com 60 anos, alcançando prevalência entre 20 a 30% na idade de 85 anos.^5^ Algumas projeções estimam que, no ano de 2020, 15% da população brasileira deve estar com mais de 60 anos, e que o Brasil venha a ser o sexto país do mundo em número de idosos em 2025.^3^

O comprometimento cognitivo leve (CCL) caracteriza-se pela queixa cognitiva relatada pelo próprio paciente e/ou familiar e por comprometimento cognitivo objetivo avaliado por testes cognitivos, baseando-se nas normas para idade e escolaridade. Além disso, as atividades funcionais devem estar preservadas.^6^ Alguns estudos consideram o CCL um estado de transição entre as mudanças cognitivas observadas no envelhecimento e a demência propriamente dita.^7^ Assim, é importante classificar o CCL em subtipos, como: CCL amnéstico de único domínio (CCL-AUD), CCL amnéstico de múltiplos domínios (CCL-AMD), CCL não amnéstico de único domínio (CCL-NAUD) e CCL não amnéstico de múltiplos domínios (CCL-NAMD).^8^ Quando o subtipo de CCL amnéstico evolui para demência, a doença de Alzheimer é a mais comum. No caso de CCL não amnéstico, é mais frequente a conversão para degeneração lobar frontotemporal ou demência com corpos de Lewy.^7^ Os dados de prevalência na literatura mundial referente ao CCL são muito variáveis, e a prevalência variou entre 3% e 42%.^9^

## OBJETIVO

Avaliar o perfil epidemiológico dos pacientes encaminhados com suspeita de comprometimento cognitivo leve ou demência para um ambulatório de referência de desordens da memória e do comportamento.

## MÉTODOS

O presente estudo foi submetido e aprovado no Comitê de Ética da Universidade Positivo sob número 1.533.211, CAAE: 55276416.0.0000.0093. Trata-se de estudo retrospectivo, realizado por revisão de prontuários, utilizando banco de dados eletrônico disponibilizado pelo Instituto de Neurologia de Curitiba, em Curitiba (PR). O estudo coletou dados dos pacientes encaminhados ao ambulatório no período entre janeiro de 2008 a dezembro de 2015.

Foram incluídos pacientes com idade ≥18 anos de idade, que preenchessem os critérios diagnósticos para demência segundo o Manual Diagnóstico e Estatístico de Transtornos Mentais, quinta edição (DSM-IV) da *American Psychiatric Association* (APA)^10^ e/ou os critérios diagnósticos para CCL.^11^

Os critérios de exclusão foram: prontuários incompletos e doenças que afetavam a cognição que não preencheram critérios para síndrome demencial ou CCL, como: transtorno do *deficit* de atenção e hiperatividade, lesão cerebral traumática, doenças do neurodesenvolvimento, comprometimento cognitivo vascular não demência (CCV-ND) e alterações cognitivas relacionadas à epilepsia.

Todos os pacientes foram avaliados por uma mesma equipe de neurologistas. A investigação realizada fazia parte do protocolo do atendimento do ambulatório e seguiu as recomendações do Departamento Científico de Neurologia Cognitiva e Envelhecimento da Academia Brasileira de Neurologia.^11^ A investigação consistia em anamnese detalhada, exame clínico e neurológico, avaliação cognitiva, exames de sangue e exame de neuroimagem − preferencialmente, ressonância magnética (RM) de encéfalo ou tomografia computadorizada de crânio (TCC), quando contraindicada a realização de RM de encéfalo.

Os dados coletados para este estudo foram sexo, raça, idade, escolaridade e dominância manual. O diagnóstico era estabelecido com base nos critérios diagnósticos utilizados na rotina do Ambulatório de Desordens da Memória e do Comportamento (ADEMEC). Assim, para cada tipo de demência, critérios diagnósticos específicos eram utilizados, como descritos a seguir: *National Institute of Neurological and Communicative Disorders and Stroke* (NINDS) e *Alzheimer’s Disease and Related Disorders Association* para DA;^12^ NINDS e *Association Internationale pour la Recherche et l’Enseignement en Neurosciences* (AIREN) para demência vascular;^13^ *Third Report of the Dementia Lewy Bodies* (DLB) *Consortium* para demência com corpos de Lewy;^14^ e critérios de Lund e Manchester para demência frontotemporal.^15^

De acordo com o diagnóstico, os pacientes incluídos foram divididos em três grupos: síndrome demenciais, CCL e outras síndromes demenciais raras.

A Escala de Avaliação Clínica (CDR - *Clinical Dementia Rating* )^16 , 17^ foi o instrumento utilizado para auxiliar no diagnóstico clínico e classificar o estágio das demências. Além disso, a CDR tem sido utilizada na prática clínica, para auxiliar a diferenciar os pacientes com CCL daqueles que evoluem para demência.^17^ Assim, escore igual a zero corresponde a não demência; 0,5 é questionável ou corresponde a CCL; 1 à demência leve; 2 à demência moderada e 3 à demência grave.

Os dados coletados foram planilhados no programa Excel^®^e a análise estatística foi realizada com o auxílio do programa *Graph Pad Prism 6.0* (La Jolly, EUA). As variáveis contínuas foram expressas como média±desvio padrão e comparadas com os testes *t* e Mann-Whitney. Variáveis categóricas foram expressas em percentagens e comparadas com o teste do χ^2^ ou teste exato de Fisher, conforme apropriado; calcularam-se os valores de p, razão de chance (RC) e do IC95%. Foram considerados significativos valores de p<5%.

## RESULTADOS

Foram avaliados os prontuários de 1.060 pacientes encaminhados para o Ambulatório de Desordens da Memória e do Comportamento no período do estudo. Dos 1.060 pacientes avaliados, 760 pacientes foram incluídos. O principal motivo para exclusão de pacientes se deveu ao fato de o ambulatório atender inicialmente pacientes com queixas cognitivas diversas. Então, somente após ter sido estabelecido o diagnóstico de desordens cognitivas não compatíveis com síndrome demencial e/ou CCL, os pacientes foram encaminhados para outros ambulatórios especializados.

Os pacientes incluídos (n=760) foram divididos em três grupos: síndromes demenciais, CCL e outras síndromes demenciais raras. Observou-se predomínio das demências, que afetaram 68,8% (523/760) dos casos estudados. O CCL foi diagnosticado em 17,4% (133/760), e os grupos de demências degenerativas raras e/ou de demências não degenerativas corresponderam a 13,8% (102/760) dos pacientes.

Os dados demográficos dos pacientes estudados estão representados na tabela 1 . Dentre os pacientes encaminhados, evidenciou-se, de maneira significativa, predomínio do sexo feminino (61,3%; p<0,0001; RC=2,5; IC95%: 2,0-3,1). A média de idade foi de 67,7±15,67 anos. Como apresentado na tabela 1 , a partir dos 60 anos, foi observado expressivo aumento do número de casos. Em relação à raça, 96,3% eram de etnia branca, e 59,5% dos pacientes tinham nível superior completo ou incompleto.

**Tabela 1:** Dados demográficos do total de pacientes estudados (n=760)

	n (%)
Sexo	
Feminino	467 (61,3)
Masculino	293 (38,5)
Idade, anos	
18-30	23 (3)
31-40	32 (4,2)
41-50	56 (7,3)
51-60	101 (12,2)
61-70	152 (20,0)
71-80	224 (29,4)
81-90	156 (20,5)
91-100	16 (2,1)
Raça	
Branco	732 (96,3)
Afrodescendente	16 (2,1)
Amarelo	7 (0,9)
Indígena	5 (0,6)
Escolaridade, anos de estudo	
Analfabetos	11 (1,3)
0	18 (2,3)
1-4	228 (30)
5-8	50 (6,5)
9-11	131 (17,2)
>11	322 (42,3)

Quando se verificou separadamente os grupos, podemos visualizar, na tabela 2 , os tipos demência encontrados. A doença de Alzheimer representou quase metade dos casos de demência (48,9%), seguida da demência vascular (11,3%) e da mista (7,8%), que se apresentaram nos pacientes com doença de Alzheimer e demência de causa vascular concomitante. Dentre os casos de demência, a distribuição entre os sexos mostrou que a doença foi mais frequente em mulheres (312/523; 59,6%) do que em homens (211/523; 40,4%), conferindo-se diferença significativa (p<0,0001; RC=2,1; IC95%=1,6-2,7). A média de idade dos pacientes no geral foi de 70,1±14,90 anos. A média de idade dos homens foi 68,5±15,63 anos e das mulheres 71,2±14,43 anos, mostrando que homens apresentaram a demência mais precocemente que as mulheres (p<0,0001).

**Tabela 2:** Classificação das síndromes demenciais quanto à sua prevalência (n=523)

Síndromes demenciais	n (%)
Doença de Alzheimer	256 (48,9)
Demência vascular	59 (11,3)
Demência mista	41 (7,8)
Hidrocefalia de pressão normal	31 (5,9)
Demência com corpos de Lewy	26 (5)
Demência não especificada	21 (4)
Paralisia supranuclear progressiva	15 (2,9)
Demência frontotemporal	12 (2,3)
Outras	62 (11,9)

Em relação à escolaridade e à idade do diagnóstico, quando se compararam as pessoas com maior escolaridade (acima de 9 anos) com aquelas de menor escolaridade (1 a 4 anos), observou-se que a idade de aparecimento do quadro de demência foi significativamente menor naqueles com maior tempo de estudo (p=0,0007). Quando comparados os de maior escolaridade (acima de 9 anos) com aqueles com escolaridade de nível médio (5 a 8 anos), não se observou diferença significativa (p=0,09). Analisando-se os que tinham entre 1 a 4 anos com aqueles entre 5 a 8 anos, também se evidenciou diferença significativa (p=0,036).

Na tabela 3 podemos notar a distribuição dos escores da CDR, que avaliou o estágio de demência dos pacientes estudados. Quando comparados doença de Alzheimer e demência vascular, não se verificou diferença significativa entre os estágios de CDR (p=0,78), demonstrando gravidade similar entre as demências mais encontradas. Relacionando a influência da escolaridade com CDR, foi possível observar que, em pacientes com menor escolaridade (menos que 9 anos de estudo), CDR teve significativamente maior pontuação quando comparado a pacientes com maior grau de escolaridade (mais que 9 anos de estudos; p<0,0001; OR=3,6; IC95%: 2,4-5,3). Ressalta-se que CDR com estágio 3 foi frequente em mais pacientes com pouca escolaridade.

**Tabela 3:** Análise dos estágios da Escala de Avaliação Clínica nos pacientes com demência estudados, comparando-se tipo de demência e escolaridade

	CDR=1	CDR=2	CDR=3
	n (%)	n (%)	n (%)
Tipo de demência			
Doença de Alzheimer (n=256)	119 (46,5)	95 (37,1)	42 (16,4)
Demência vascular (n=59)	24 (40,7)	28 (47,4)	7 (11,9)
Outras (n=208)	84 (40,7)	82 (39,4)	42 (19,9)
Escolaridade*			
Até 9 anos (n=196)*	57 (29,0)	89 (45,4%)	50 (25,5)
Acima de 9 anos (n=263)	157 (59,7)	79 (30,0)	26 (9,8)

* Comparando-se grau de escolaridade *versus* CDR=3 (p<0,0001; RC=3,6; IC95%: 2,4-5,3). CDR: Escala de Avaliação Clínica.

O estadiamento da demência pela CDR também possibilitou observar que, no momento do diagnóstico, 46,5% dos pacientes apresentavam estágio 1, 37,0% estágio 2 e 16,5% dos casos estágio 3 da CDR.

Em relação aos pacientes diagnosticados com CCL, na tabela 4 , observamos os tipos de CCL encontrados. Dentre os cinco subtipos de CCL, que se diferenciaram pelo tipo de domínios afetados, o subtipo CCL-AUD foi o mais prevalente, representando 77,3% dos casos encontrados. No que diz respeito à idade dos pacientes com CCL, a média foi de 61,7±14,82 anos, sendo a idade dos homens 59,1±15,74 anos e das mulheres 62,2±16,02 anos. Ao comparar a média de idade, não houve diferença na idade de aparecimento do CCL entre homens e mulheres.

**Tabela 4:** Classificação dos pacientes com comprometimento cognitivo leve, conforme o subtipo encontrado (n=133)

Tipos de CCL	n (%)
CCL-AUD	103 (77,3)
CCL-AMD	18 (13,6)
CCL-NAMD	6 (4,5)
CCL DPI	2 (1,5)
CCL-NAUD	2 (1,5)
Comprometimento cognitivo a esclarecer	2 (1,5)

CCL: comprometimento cognitivo leve; AUD: amnésico de único domínio; AMD: amnésico de múltiplos domínios; NAMD: não amnésico de múltiplos domínios; DPI: doença de Parkinson idiopática; NAUD: não amnésico de único domínio.

Com relação à escolaridade, os pacientes com mais anos de estudo (≥9 anos) apresentaram CCL significativamente mais jovens quando comparados aos com menos de 9 anos de escolaridade (p=0,03).

## DISCUSSÃO

O presente estudo apresenta de forma original a epidemiologia de uma robusta amostra de pacientes brasileiros com demência e CCL, atendidos somente por planos de saúde ou particular, em um centro de referência na Região Sul do Brasil. Este ainda tem como diferencial apresentar alta escolaridade e renda, quando comparado ao restante da população do nosso país.

Em relação ao sexo, observamos maior prevalência de demência entre as mulheres, sendo duas vezes mais frequente que nos homens, corroborando dados obtidos por outros autores.^1 , 3^ Uma possível explicação para a maior prevalência de demência em mulheres pode ser a expectativa de vida. Adicionalmente, fatores como maior exposição ao estresse do mercado de trabalho, abuso do álcool e cigarro, também podem ter papel relevante no maior acometimento de demência nas mulheres.^3^ Por outro lado, Bottino et al., não demonstraram diferença na prevalência de demência entre homens e mulheres.^18^

Quanto à classificação, em relação à idade, a demência de início precoce (pré-senil), considerada pelo início dos sintomas cognitivos em pessoas com menos de 65 anos, foi mais frequente nos homens. Alguns estudos também demonstraram o predomínio de demência de início precoce no sexo masculino.^19 , 20^ Hábitos de vida classicamente mais associados com o sexo masculino, como etilismo e tabagismo, entre outros, podem influenciar em tal achado.

No presente estudo, entre todos os pacientes diagnosticados com demência ou CCL, foi expressivo o aumento na prevalência nas faixas etárias de 61 a 70 anos (de 20%), 71 a 80 (de 29%) e 81 a 90 (de 20%). Entretanto, em idosos entre 91 a 100 anos, foi de 2%. Alguns estudos com pacientes em idades muito avançadas, acima de 90 anos, sugerem a ocorrência de um platô, mas o número de idosos com mais de 90 anos no nosso estudo era muito pequeno para permitir tal avaliação. Tal associação entre a idade e o aumento na prevalência de demência ou CCL também foi encontrada por outros autores na população brasileira.^18 , 21 , 22^

Neste estudo, a demência afetou a etnia branca em 96,3% dos casos. Lopes et al.,^23^ não mostraram diferenças na prevalência de demências entre negros e brancos. Ressaltamos que a desigualdade na distribuição de renda vigente em nosso país confere maior acesso das pessoas de etnia branca ao atendimento privado, e Região Sul foi colonizada predominantemente por caucasianos − fato que não reflete a realidade na maior parte do Brasil.

Maior escolaridade do paciente esteve significativamente associada com o aparecimento mais precoce da demência. Tal achado é discordante da literatura.^18 , 22 , 24^ Na população brasileira, pessoas com baixa escolaridade têm maior risco de demência, chegando a ser seis vezes maior em analfabetos.^18 , 22 , 24^ Nossa pesquisa foi realizada em um hospital privado, com um grupo de pacientes de maior escolaridade e renda do que a população brasileira em geral − apenas 1,1% dos pacientes eram analfabetos. Acreditamos que apresentamos um perfil de brasileiros da classe média, cujos os dados podem diferir dos observados em estudos realizados com pacientes atendidos pelo Sistema Único de Saúde (SUS). Sugere-se, ainda, que os pacientes com maior escolaridade realizam atividades diárias diversificadas e estejam sujeitos a uma maior demanda cognitiva, sendo mais facilmente perceptíveis a pequenas mudanças cognitivas. Além disso, a maior renda confere acesso facilitado e mais rápido aos ambulatórios que atendem tal demanda, fato que também pode proporcionar resultados diferentes dos encontrados na literatura.

Como já era esperado, a doença de Alzheimer foi a forma mais comum de demência entre nossos pacientes (48,9%), estando de acordo com o descrito por outros autores.^25^ Rizzi et al.,^1^ descreveram que a doença de Alzheimer representou 60% e a demência vascular 20% dos casos de demência em diversas regiões do mundo.

Não houve diferença no estágio da CDR quando se compararam os tipos de demências mais frequentes. No entanto, dado que diferenciou o perfil do ambulatório privado deste estudo é que quase metade dos pacientes diagnosticados com demência apresentavam CDR 1 (leve) e 2 (moderado), e 16,5% procuraram o serviço com CDR estágio 3 (grave). A maioria dos pacientes procurou o atendimento especializado em fases iniciais da demência. Esse fato é de grande importância, já que o diagnóstico precoce pode implicar em evolução mais favorável da doença, quando adotadas medidas, como: controle adequado de fatores de risco cardiovasculares; mudança no estilo de vida, como a prática de atividade física, abstenção de certos medicamentos prejudiciais para a cognição, abuso de álcool, tabagismo e reabilitação cognitiva; e tratamento farmacológico precoce. Além disso, o diagnóstico precoce da doença permite que o paciente, os familiares e os cuidadores estejam mais preparados para lidar com a doença e sua evolução.^1 - 4^

Foram diagnosticados em estágio 3 da CDR três vezes mais pacientes com menor escolaridade do que com maior escolaridade. Tal fato pode ser devido à maior demora das pessoas com menor escolaridade terem acesso ao serviço especializado. Estudo realizado no Rio Grande do Sul^26^ utilizando o mesmo escore não encontrou influência da escolaridade dos pacientes na classificação da CDR.

Os dados de prevalência na literatura mundial referente ao CCL são muito variáveis, por conta de diferentes critérios diagnósticos utilizados.^9^ O CCL foi diagnosticado em 17,4%, com média de idade de 62,2 anos, mais precoce que nos quadros de demência. Petersen et al., descreveram que a prevalência de CCL foi semelhante à encontrada em nosso estudo, variando entre 12 a 18%.^27^

O CCL tal como nas demências também foi mais frequente no sexo masculino do que no feminino. Ainda de maneira similar ao observado nas demências, o CCL esteve presente mais cedo de forma significativa em pacientes com maior escolaridade (≥9 anos). Dentre os quatro principais subtipos de CCL descritos, o amnéstico é mais frequente, embora se observe divergência de resultados na literatura mundial.^28^ Cabe ressaltar que há dificuldade para se compararem estudos de prevalência de CCL, visto que as diferenças nos achados podem ser devidas à utilização de diferentes critérios diagnósticos e à influência de fatores como idade, escolaridade, instrumentos utilizados e genética da população estudada.

A limitação da nossa pesquisa se deve ao próprio desenho retrospectivo, no qual as informações coletadas são oriundas de prontuários, o que não permite conhecer o desfecho evolutivo da população estudada. Tal informação seria de grande relevância, já que não permite estudar uma população de pacientes denominada com comprometimento cognitivo não demência *.* O conceito de comprometimento cognitivo não demência engloba pacientes com CCL e indivíduos com desempenho cognitivo abaixo do esperado para a idade e a escolaridade, que podem ou não declinar.^29 , 30^ A força do presente estudo reside no tamanho da amostra e no perfil diferenciado da casuística composta por pacientes de uma instituição privada e com maior nível educacional, fornecendo dados relevantes sobre uma população muito pouco estudada em nosso país.

## CONCLUSÃO

Maior parte dos pacientes avaliados tinha o diagnóstico de demência entre todas as doenças atendidas. A síndrome demencial mais frequente foi a doença de Alzheimer. Em relação ao comprometimento cognitivo leve, o subtipo amnésico de único domínio foi o mais prevalente. As mulheres foram duas vezes mais acometidas que os homens, mas os homens apresentaram o quadro demencial significativamente mais jovens. Pacientes com maior escolaridade foram diagnosticados significativamente mais jovens, tanto em relação à demência quanto ao comprometimento cognitivo leve, que aqueles com menor escolaridade. Contudo, pacientes com menor escolaridade apresentaram maior comprometimento cognitivo.
